# Effect of temperature and relative humidity on the development times and survival of *Synopsyllus fonquerniei* and *Xenopsylla cheopis*, the flea vectors of plague in Madagascar

**DOI:** 10.1186/s13071-016-1366-z

**Published:** 2016-02-11

**Authors:** Katharina S. Kreppel, Sandra Telfer, Minoarisoa Rajerison, Andy Morse, Matthew Baylis

**Affiliations:** LUCINDA group, Institute of Infection and Global Health, Department of Epidemiology and Population Health, University of Liverpool, Leahurst Campus, Neston, CH64 7TE UK; School of Biological Sciences, University of Aberdeen, Tillydrone Avenue, AB24 2TZ Aberdeen, Scotland UK; Unité Peste - Institut Pasteur de Madagascar, BP 1274, Antananarivo, 101 Madagascar; Department of Geography and Planning, School of Environmental Sciences, University of Liverpool, Liverpool, Merseyside L69 3GP UK; Health Protection Research Unit in Emerging and Zoonotic Infection, University of Liverpool, Liverpool, Merseyside L69 3GP UK

**Keywords:** *Xenopsylla cheopis*, *Synopsyllus fonquerniei*, Plague vector, Development time, Degree-days, Saturated salt solutions

## Abstract

**Background:**

Plague, a zoonosis caused by *Yersinia pestis*, is found in Asia, the Americas but mainly in Africa, with the island of Madagascar reporting almost one third of human cases worldwide. In the highlands of Madagascar, plague is transmitted predominantly by two flea species which coexist on the island, but differ in their distribution. The endemic flea, *Synopsyllus fonquerniei,* dominates flea communities on rats caught outdoors, while the cosmopolitan flea, *Xenopsylla cheopis*, is found mostly on rats caught in houses. Additionally *S. fonquerniei* seems restricted to areas above 800 m. Climatic constraints on the development of the two main vectors of plague could explain the differences in their distribution and the seasonal changes in their abundance. Here we present the first study on effects of temperature and relative humidity on the immature stages of both vector species.

**Methods:**

We examined the two species’ temperature and humidity requirements under experimental conditions at five different temperatures and two relative humidities. By employing multivariate and survival analysis we established the impact of temperature and relative humidity on development times and survival for both species. Using degree-day analysis we then predicted the average developmental threshold for larvae to reach pupation and for pupae to complete development under each treatment. This analysis was undertaken separately for the two relative humidities and for the two species.

**Results:**

Development times and time to death differed significantly, with the endemic *S. fonquerniei* taking on average 1.79 times longer to complete development and having a shorter time to death than *X. cheopis* under adverse conditions with high temperature and low humidity. Temperature had a significant effect on the development times of flea larvae and pupae. While humidity did not affect the development times of either species, it did influence the time of death of *S. fonquerniei*. Using degree-day analysis we estimated an average developmental threshold of 9 °C for *S. fonquerniei*, and 12.5 °C for *X. cheopis*.

**Conclusions:**

While many vector-borne diseases are limited to warm, low-lying regions, plague in Madagascar is unusual in being most prevalent in the cool, highland regions of the country. Our results point towards the possibility that this is because the endemic flea vector, *S. fonquerniei,* is better adapted to cool temperatures than the exotic flea vector, *X. cheopis.* Future warming caused by climate change might reduce the area suitable for *S. fonquerniei* and may thus reduce the incidence of plague in Madagascar.

## Background

Plague, a vector-borne, highly virulent zoonotic disease is still present today in Africa, Asia and the Americas. It is caused by infection with the bacterium *Yersinia pestis,* with rodents and their fleas as its principal hosts. Transmission of bubonic plague to humans occurs via the bite of an infected flea and if left untreated, it triggers serious illness with up to 55 % case fatality in human populations [1, 2]. In many parts of the world climate is known to affect plague dynamics [3–6] and like many vector borne diseases, the primary mechanisms are thought to be driven by local variation in factors such as temperature and rainfall. Presently, Africa accounts for more than ninety percent of all human plague cases reported globally. Within African countries, the majority of cases are reported from Madagascar and the Democratic Republic of Congo [1]. With 482 notified cases in 2014, Madagascar has for many years been the country worst affected by plague, experiencing seasonal recrudescence between September and April [7].

Plague is endemic in the highland region of Madagascar, above 800m of altitude. The reasons for such pronounced foci include unique host and vector dynamics, special climate features such as warm, wet austral summers and cool, dry winters as well as extreme poverty [8]. In Madagascar, the principal host appears to be the black rat, *Rattus rattus* [9], with two flea species primarily involved in plague transmission: *Xenopsylla cheopis (X. cheopis)*, a cosmopolitan, exotic species, and *Synopsyllus fonquerniei (S. fonquerniei)*, an endemic species. The two vector flea species coexist on the island, but occupy different niches, with *X. cheopis* found primarily on black rats within dwellings, while the endemic *S. fonquerniei* dominates the flea communities infesting rats living outdoors [10]. Below 800 m altitude *S. fonquerniei* appears to be absent [8, 11].

In the central highlands of Madagascar, both human plague incidence and the abundance of the two flea vector species show seasonal cycles. Most human cases of plague are reported during the warm rainy season from October to March [2, 12]. At least in urban areas, numbers of adult *X. cheopis* are low from April to December, whilst in rural areas numbers of adult *S. fonquerniei* increase during the cold dry season (from July onwards), peaking in September to January [13].

Like other ectoparasite species, flea distribution and abundance can be significantly affected by climatic variables. The immature stages of fleas develop in host burrows and are sensitive to air temperature and humidity, with effects on both development and survival times [14, 15]. Thus, the regional and local differences in the spatial distribution of *X. cheopis* and *S. fonquerniei* within Madagascar, and the contrasting seasonal cycles may be driven by differences in climate susceptibility between the two species. The climate across Madagascar is described as unimodal tropical, with a hot rainy season from November to March and a cold dry season from April to October. To determine the temperature and relative humidity ranges for the experiment, information from micro-climate measurements inside burrows of the black rat (*Rattus rattus*) in the highlands of Madagascar was used. Previously published work by Sharif et al. on the development time of *X. cheopis* from India in 1949 in relation to temperature and humidity [16] was also considered to make informed decisions on the temperatures chosen for the experiment. Although some experimental data is available for *X. cheopis*, little is known about the biology of *S. fonquerniei*, and there is a critical need for information on the basic developmental constraints of the two flea vector species to provide further insight into plague epidemiology in Madagascar. Here we present the first study investigating the temperature and humidity needs of *S. fonquerniei* under experimental conditions, by examining larval and pupal development as well as survival*.* We provide original data on the effects of climate on the development and survival of the endemic *S. fonquerniei* and its counterpart *X. cheopis.*

## Methods

### Rearing conditions

Colonies of both vector species originated from adult fleas collected in 2001 from a total of 12 different highland regions of Madagascar. New specimens of both species from these regions were added every year until 2006. Since collection, they have been maintained in insectaria at the Medical Entomology unit, Institut Pasteur de Madagascar (IPM).

The fleas were reared in covered glass jars, 1/4 filled with a 50:1 mixture of rice husk and dried beef blood for larval nutrition, at 27 ± 2 °C and 70 % ± 5 % relative humidity (RH). To obtain sufficient numbers of larvae for the experiment, recently fed, adult male and female fleas were transferred into glass jars containing 2 g of rice husk as a surface for oviposition. After 24 h the adults were carefully removed and the jars with the eggs were left to stand in the insectaria for 6 days. The jars were checked once a day until newly hatched larvae were detected; larvae were only collected once from each jar to ensure the same age of the specimens. In other flea species, the RH conditions under which the eggs are incubated do not affect the development time of larvae subsequently exposed to different conditions [14].

### Experimental set up

Between 2008 and 2009, Easy Log data loggers (EL USB-2, Lascar Electronics) were used to record temperature and relative humidity inside rat-burrows (*n* = 56) during the wet and dry season in villages reporting plague. In the dry season recorded burrow temperatures ranged from 17–22 °C and 65–81 % relative humidity while in the wet season burrow temperatures were between 23–28 °C and 84–94 % relative humidity. According to this information experiments were undertaken using incubators (NUVE ES 120, UK and Binder KBF, Germany) at five temperatures (18, 21.5, 25, 28.5 and 32 °C), each at two RHs, 80 % and 90 %. Using a method described in detail by Winston and Bates [17] and widely used since [18, 19], the RHs were obtained by adding saturated salt solutions of ammonium sulphate, (NH_4_)SO_4,_ and sodium carbonate, NA_2_CO_3,_ to 1.5 cm depth within airtight plastic boxes.

With five temperatures and two RHs, there were ten experimental treatments for each flea species. RH and temperature readings were taken in each box 24 h before use, and intermittently during the experiment, with hygrometers (433MHz Cable free Hygrometer, Oregon Scientific, UK) to ensure that the climatic conditions were maintained.

Newly hatched *S. fonquerniei* (*n* = 1,288) and *X. cheopis* (*n* = 750) larvae, up to 24 h old, were transferred onto halved ELISA*-*plates (NUNC MaxiSorp™ polystyrene 96 well), with one larva per well. Each well contained 5 mg of larval rearing medium (powdered rodent diet, dried beef blood and dried yeast at a ratio of 20:3:1 respectively). As a higher mortality rate became apparent, more *S. fonquerniei* larvae were reared than *X. cheopis*. Plates were placed on stands in the airtight boxes within the incubators and covered with fine nylon mesh to prevent escape during the first two days when the larvae are most active.

Larval survival and cocoon formation (also referred to as pupation) were noted daily. The developmental growth progress of the larval stages of both species was recorded every second day by placing the transparent ELISA plate under a microscope lit from below. Pupation indicated completion of the active larval stage. Larvae were observed until they either pupated or died, except for a small number which had still not pupated after twice the time taken by the majority of their counterparts; these individuals were assumed to have a developmental problem and, although still alive, they were excluded from the study population (2.74 %).

After pupation, cocoons were individually transferred into 5 ml Eppendorfs and sealed with fine nylon mesh to prevent the adult flea escaping, once hatched. Each cocoon was then subjected to the same conditions as the larva before pupation. In some cases, however, cocoons were attached to the well, and could not be removed without risk of damage. In these cases, the wells were sealed with perforated Parafilm and maintained as for those in Eppendorfs. Eppendorfs and plates with cocoons were shaken gently every day to help stimulate emergence [20]. Once emerged, the adults were immobilised using ice to enable species confirmation and sex identification.

### Statistical analysis

#### Flea development: time to pupation and time to emergence

The data were divided into two sets for analysis: larval development time to pupation (LDT) and pupal development time to emergence (PDT). These analyses were restricted, respectively, to larvae that pupated successfully (521/750 (69.5 %) of *X. cheopis* and 245/1288 (19.0 %) of *S. fonquerniei*), and to pupae that emerged as adults (258/521 (49.5 %) of *X. cheopis* and 77/245 (31.4 %) of *S. fonquerniei*). Temperature was log-transformed. LDTs and PDTs were tested for normal distribution using an Anderson Darling test. Outliers greater than the mean ± 2 SD were removed from the analysis. For each dataset, linear mixed effects models were first used to examine the effects of temperature and RH on development time of each species individually. There was a potential of non-independence of samples, as the individual larvae and pupae of one plate were likely to be from the same batch of eggs. To account for possible non-independence, *plate* was included as a random effect in all models. Subsequently, models were developed for the data of both species combined, with *species* as a fixed effect, and two-way interaction terms between temperature, RH and *species*. The purpose of the combined model was to examine differences in the effects of RH and temperature between species. Due to heterogeneity of variance in LDTs and PDTs between temperature treatments, a fixed variance structure as a function of temperature was used by applying a weights argument. This accounted for the larger variance in development times at lower temperatures. Model selection was based on the Akaike information criterion (AIC) [21] and the principle of parsimony: only when the AIC was reduced by 2 or more was the inclusion of an additional variable considered an improvement to the model [22]. All statistical analysis was carried out using the nlme package [23] in R 3.1.2 for Windows [24].

#### Mortality and time of death

To investigate the effect of species, temperature and humidity on the timing of death, survival analysis [25] was applied to the dataset on larval development. Pupal survival was not studied, as the time of death within their cocoon could not be established. Analysis was based on a Cox proportional hazard model. Model selection was based on AIC values. Larvae without a recorded time of death (i.e., remained as larvae, or had successfully pupated by the end of the experiment) were right-censored. Survivorship over time was plotted as Kaplan Meier survival curves for each treatment and species.

#### Degree-day analysis

A widely used method, degree-day analysis, was chosen to determine the thermal units needed by larvae to reach pupation, and by pupae to complete development [26, 27]. This analysis was undertaken for each species at the two RHs separately. Development rate is the reciprocal of development time. As development rates plateaued at the highest temperature in both species, a regression line was fitted to the linear part of the observed data, thus omitting data at 32 °C. The linear regression equation was extrapolated to the x-intercept to estimate the lower developmental threshold—the temperature at which the development rate is zero and the development time would therefore be infinite. The reciprocal of the slope of the linear regression was then used to estimate the thermal constant (k-value) of each species [28]. The K-value defines the sum of thermal energy above the threshold required for 50 % of the population to complete their development stage. The thermal constant is expressed in degree-days (DD).

## Results

Analysis of the raw data revealed that more individuals of *X. cheopis* completed both development stages successfully and emerged as adults than the endemic *S. fonquerniei*. Sixty-nine percent of larval *X. cheopis* survived to pupation, and 34 % to adulthood. By comparison, only 19 % and 6 % of *S. fonquerniei* larvae reached the pupal and adult stages respectively. The male: female ratio of the emerged *X. cheopis* was 1: 0.79; that of *S. fonquerniei* was 1:2.5.

### The effects of treatment on development time

**Fig. 1:**
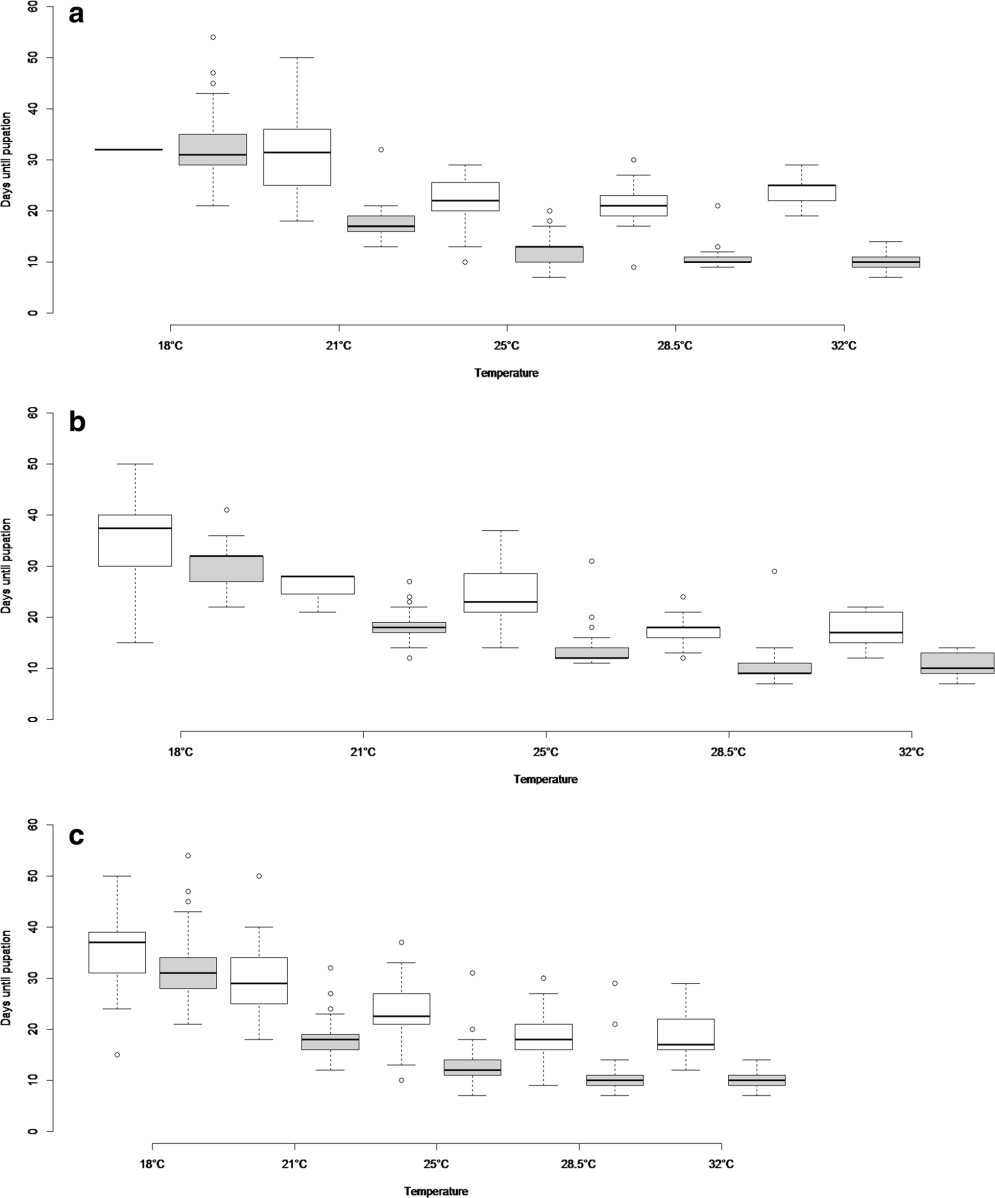
Distribution of larval development times (LDT) for *S. fonquerniei* (white) and *X. cheopis* (grey) at each temperature showing median values with 95 % confidence intervals and first and third quartile. **a** 80 % relative humidity **b**) 90 % relative humidity **c** both humidities combined

The distributions of LDT and PDT were not significantly different from normal. LDTs of *X. cheopis* were faster than *S. fonquerniei* at almost all temperatures, at both 80 % (Fig. 1a) and 90 % RH (Fig. 1b); the single exception was at 18 °C, 80 % RH, where *S. fonquerniei* developed at a similar rate to *X. cheopis*. At both RHs, the difference between the species was smallest at 18 °C. Pooling data for both RHs, likelihood ratio tests confirmed the LDTs were significantly different between species at every temperature (Fig. 1c).

Considering each species separately, LDT for both species was best explained by temperature combined with the random effect *plate.* In both cases, LDT was negatively associated with temperature (Table 1a). In each of the best single species models RH had no effect on LDT.

**Table 1:** Results of the best model for development time for each vector species separately. Model selection is based on ΔAIC and parsimony. For both species variables include temperature (logtemperature) and PCR plate (plate)

a) Larval development time until pupation				
Larval development time				
	*Model: temperature (log), plate*			
Species	Variable	coeff	SE	*p*-value
*X. cheopis*	Intercept	127.05	12.34	<0.0001
	Logtemperature	−79.67	8.90	<0.0001
*S. fonquerniei*	Intercept	107.77	13.47	<0.001
	Logtemperature	−59.79	9.55	<0.001
b) Pupal development time until emergence				
Pupal development time				
	*Model: temperature (log), plate*			
Species	Variable	coeff	SE	*p*-value
*X. cheopis*	Intercept	145.10	14.75	<0.0001
	Logtemperature	−88.82	10.58	<0.0001
*S. fonquerniei*	Intercept	87.16	15.28	<0.0001
	Logtemperature	−49.47	10.68	0.0002

**Table 2:** Results of the model comparison for development time for both vector species combined for larval development time until pupation and for pupal development time until emergence

*X. cheopis and S. fonquerniei*				
	Larvae		Pupae	
Model	df	Δ AIC	df	Δ AIC
RH + species, plate	5	4381	5	2253
Logtemperature + species, plate	5	4326	5	2210
Logtemperature + RH + species, plate	6	4327	6	2208
Logtemperature | RH + species, plate	7	4328	7	2208
RH | species + logtemperature, plate	7	4322*	7	2208
Logtemperature | species, plate	6	4327	6	2207
Logtemperature | species + RH, plate	7	4328	7	2205*

Plus (+) corresponds to addition of variables while slash (|) corresponds to an interaction term between variables. Model selection is based on ΔAIC and parsimony. The selected models are marked with an asterisk

Considering both species together, the best model for LDT, on the basis of the smallest AIC, combined temperature, RH, species and plate. In this model (Table 3a), the main effects for RH, temp and species, and the interactions of species and RH, were all significant.

**Table 3:** Results of the best model for development time for both vector species combined. Model selection is based on ΔAIC and parsimony

a) For larval development time until pupation. Variables include humidity (RH %), species, temperature (logtemperature) and PCR plate (plate).			
*X. cheopis and S. fonquerniei*			
LARVAE			
*Model: RH % | species + temperature (log), plate*			
Variable	coeff	SE	*p*-value
intercept	126.1829	20.989	0.0003
logtemperature	−71.898	14.813	0.0156
RH 90 %	−3.4024	26.453	0.0242
species *X. cheopis*	−9.3341	22.636	0.0047
species *X. cheopis* | RH 90 %	3.37912	18.673	0.0200
b) For pupal development time until emergence. Variables include species, temperature (logtemperature) and PCR plate (plate).			
*X. cheopis and S. fonquerniei*			
PUPAE			
*Model: species | temperature (log) + RH %, plate*			
Variable	coeff	SE	*p*-value
intercept	88.07774	19.35798	
logtemperature	−48.8746	13.57655	0.0009
species *X. cheopis*	55.22215	22.76055	0.0159
RH 90 %	−2.61925	1.378665	0.0649
logtemperature | species *X. cheopis*	−37.7761	15.97802	0.0187

Patterns for PDT were broadly similar to LDT. Considering each species separately, PDT for both species was best explained by temperature combined with the random effect *plate.* In both cases, PDT was negatively associated with temperature (Table 1b). There was no effect of RH on PDT in either species.

Considering both species together, the best model for PDT again combined temperature, RH, species and plate (Table 2). Two variables (temperature and species) were significant, as was the interaction between them (Table 3b), while RH was not.

### Survival analysis

**Fig. 2:**
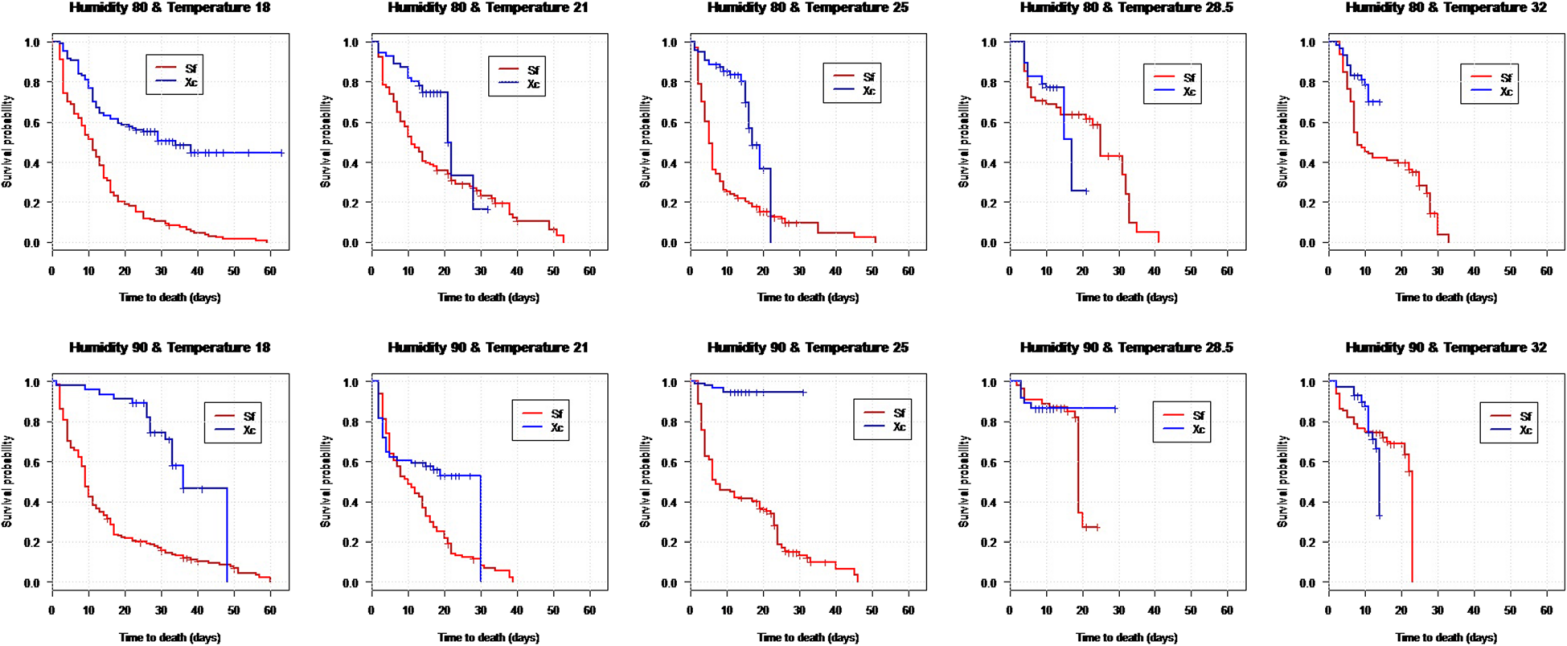
Kaplan-Meier survival curves by species showing the survival probability for larvae across time at different humidities. The blue line shows the survival probability decline for *X. cheopis* while the red line shows the decline of the survival probability of *S. fonquerniei*

Overall the Kaplan Meier survivorship curves indicated that *S. fonquerniei* larvae showed a higher mortality and faster decline in survivorship than *X. cheopis* (Fig. 2). In addition, the curves indicated that at the lower three temperatures the survival for *S. fonquerniei* declined faster under both RHs than the survival of *X. cheopis*. This was also valid at 32 °C temperature under 80 % RH, while at 28.5 °C for both RHs and at 32 °C with 90 % RH, survival declined in a similar pattern in both species. In the Cox Proportional Hazards model, time to death of *X. cheopis* was significantly influenced by RH only, with larvae tending to live longer at higher RH (Table 4; Fig. 2). The effect of temperature and the interaction between temperature and RH were not significant. For *S. fonquerniei*, however, both RH and the interaction of RH with temperature significantly influenced survival time, while the effect of temperature alone approached but did not reach significance. Considering the significant interaction, at higher temperatures larvae lived longer at 90 % than 80 % RH, but at lower temperatures there was little effect of RH (Fig. 2). The model for both species combined showed a significant effect of species (β = −1.176, 95 %, *P* < 0.0001, approximate hazard ratio = 0.30, CI = 0.26–0.35) and a significant interaction between temperature and RH (β = −0.045, 95 %, *P =* 0.0001, approximate hazard ratio = 0.95, CI = 0.93–0.97).

**Table 4:** Results from the Cox proportional hazard analysis on time to death for *X. cheopis* and *S. fonquerniei*

	Variable	Coefficient	95 % CI	*P*-value	Approx. hazard
*X. cheopis*	Temperature	0.01	0.97–1.04	0.56	1.01
	Relative humidity 90 %	−0.347	0.52–0.94	0.018	0.7
	Temperature | Relative humidity	−0.016	0.92–1.04	0.57	0.98
*S. fonquerniei*	Temperature	−0.016	0.96–1.00	0.062	0.98
	Relative humidity 90 %	−0.271	0.67–0.86	<0.0001	0.76
	Temperature | Relative humidity	−0.046	0.93–0.97	0.0003	0.95

Temperature was included as a continuous variable while relative humidity was considered dichotomous. Hazard values are estimated hazard values, calculated by taking the exponential of the coefficient

**Table 5:** Thermal units needed by flea vector larvae of *X. cheopis* and *S. fonquerniei* to complete development to pupae at two humidities combined and for 80 % RH and 90 % RH separately

		80 % + 90 %	80 %	90 %
*X. cheopis*	Threshold	12.36	12.34	12.71
	R ^2^ - value	0.77	0.75	0.76
	K - value	158.15	158	155
*S. fonquerniei*	Threshold	9.26	9.14	8.57
	R ^2^ - value	0.45	0.40	0.47
	K - value	361.6	360.77	345

### Degree-day analysis

Too few *S. fonquerniei* larvae developed at 18 °C to include them in this analysis. At 32 °C the development time plateaued and thus the data was also excluded. Using the remaining 3 temperatures, the developmental threshold (intercept of the regression line and the x-axis) was 12.4 for *X. cheopis* and 9.3 for the endemic *S. fonquerniei,* a difference of 3.1 °C (Table 5). The number of thermal units (k-value) needed by 50 % of *X. cheopis* to complete larval and pupal development was 158 and 425 degree-days; while for *S. fonquerniei* it was 362 and 682 respectively*.*

## Discussion

The study revealed longer larval and pupal development times with higher mortality rates for the endemic plague vector *S. fonquerniei* than *X. cheopis*. We also found a lower thermal developmental threshold for the former than for the latter. For both species temperature affected larval and pupal development times significantly, while the relative humidities included in this study did not. However, higher humidity reduced the risk of death at higher temperatures in *S. fonquerniei*.

### Larval and pupal development times

*X. cheopis,* a widely distributed and well-studied flea species*,* was found to prefer higher temperatures in the laboratory. This is consistent with results from Sharif [16] and Mellanby [29]. There were differences between the species in the effects of temperature and humidity on development time. Temperature was the dominant factor, influencing the development rate of juvenile stages of both species. At higher temperature an accelerated development of larvae and pupae was observed in both species. Within the limitations of the study the effect of humidity was less prominent, with an effect only found on larval development when looking at both species together. The effect of only two humidities was tested and lower RH was associated with shorter development time for both species in combination with temperature. Larvae of *S. fonquerniei* developed much slower than the larvae of *X. cheopis* at all temperatures apart from the lowest (18 °C). Pupal development time was similar for the two species, but, as with larvae, pupal development in *X. cheopis* was significantly more sensitive to declining temperatures.

### Mortality and time to death

There were also differences between the species in the effects of temperature and humidity on survival time. Under laboratory conditions, larvae of *X. cheopis* survived longer at higher relative humidity, but no effect of temperature was detected. By contrast, survival times of larvae of *S. fonquerniei* were unaffected by relative humidity at low temperature, but at higher temperature they survived longer at higher relative humidity. Therefore, both species appear to survive longer at higher RH, but this is only true for *S. fonquerniei* when at higher temperature. This is consistent with an effect of desiccation on survival of the larvae of *S. fonquerniei.* Air is capable of holding more moisture at higher temperatures, and warmer, dry air is therefore more able to desiccate insects than cooler, dry air. This effect of increased temperature as a cause of desiccation and hence mortality is partially offset at higher relative humidity, as the warm air’s ability to desiccate is reduced.

### Degree-days

Converting the development times to development rates, and plotting the rates against temperature, indicates that the temperature threshold for development is substantially lower for *S. fonquerniei* than *X. cheopis*. This is perhaps not unexpected; given the restricted distribution of *S. fonquerniei’s* to the cool highlands of Madagascar, while *X. cheopis* is found throughout the island, and elsewhere. This may also be consistent with the presence of *S. fonquerniei* in outdoor rat burrows, while *X. cheopis* tends to be found only in burrows in houses which may be presumed to be warmer. Nevertheless, although *S. fonquerniei* is able to start developing at a lower temperature than *X. cheopis*, it requires a larger number of degree-days to achieve the same level of development (i.e. pupation).

### Implications for the field

In terms of fleas, the endemic species, *S. fonquerniei,* is found predominantly in the cooler highland regions of Madagascar [12]. Our results may provide an explanation for this. The larvae of *X. cheopis* developed significantly faster than *S. fonquerniei* in all treatments except at the lowest temperature. Our results point towards the possibility that the endemic *S. fonquerniei* is better adapted to colder conditions found in the plague regions of Madagascar, while it may be outcompeted by *X. cheopis* in the warmer conditions that prevail in other parts of the island. This is supported by the degree-day analysis, estimating the developmental threshold of *S. fonquerniei* to be 3.1 °C lower than that of *X. cheopis* and thus giving the endemic *S. fonquerniei* an advantage in the colder highlands of Madagascar. However, due to the low number of larvae pupating at 18 °C, particularly for *S. fonquerniei*, there was only limited power to estimate lower development rates and thresholds and compare them between species.

The highland plague focus in Madagascar may be due to interplay between the two vector-species present above 800 m. *S. fonquerniei’s* greater vectorial capacity [30] and lower developmental temperature threshold would enable it to sustain the plague cycle throughout the cold season where temperatures fall below 18 °C inside rat burrows until the population of *X. cheopis* is thriving again in the warm summer months.

Stenseth et al. [3] suggest that high flea abundance observed in a plague focus in Kazakhstan in spring is mediated by shorter development times, higher fecundity and increased survival due to favourable climatic conditions. Shorter development times and higher flea survival are also likely to lead to greater flea abundance in Madagascar; if so, our results suggest that greater flea abundances should occur in warmer, more humid environments, with the warmer temperatures favouring shorter development times and the higher humidity favouring survival. It is noteworthy, therefore, that the plague foci in Madagascar are largely restricted to the cool highland regions of the island [12], suggesting some disconnection between conditions favouring fleas and those favouring plague.

A frequently cited impact of climate change is that increasing temperatures will enable many vector-borne diseases to spread from warm, low-lying areas to higher, upland regions that were previously too cold. There is a considerable body of evidence that climate change has enabled malaria, for example, to spread into highland areas of East Africa and South America [31]. Plague in Madagascar, however, is unusual amongst vector-borne diseases in that it is endemic in the cool, highland areas of the country and largely absent (with the exception of the coastal town of Mahajanga) in the warm, low lying and coastal regions [32]. If this distribution is determined by non-climatic factors, such as human population density, then it is unlikely that climate change will have a significant future impact. Our study provides results that are, however, consistent with climate playing a major role in determining the distribution of plague in Madagascar; namely, the endemic flea vector *S. fonquerniei*, which has a high vectorial capacity for plague [30], survives better than the introduced flea species *X. cheopis* in the coolest, highland parts of the country. This is also supported by findings of the highest abundance of *S. fonquerniei* in the cool and dry season [2]. In that case, it may be that climate change will lead to a reduction in the incidence of plague in Madagascar as highland areas warm and become less suitable for *S. fonquerniei* and/or more suitable for *X. cheopis*.

The main aim of this study was to gather basic knowledge of the climatic constraints on the development of the flea *S. fonquerniei,* an important plague vector in Madagascar and a species endemic to that island. Previous studies on rodent fleas report species-specific effects of humidity and temperature on development times and adult survival [15, 16]. This study presents a first attempt to quantify the effects of temperature and humidity on the developmental stages of *S. fonquerniei* and compare them to those of *X. cheopis*. The range of temperatures and humidities used in this study was narrow and further laboratory work would be necessary to determine both the upper and lower developmental threshold temperatures for both species.

## Conclusions

The results of this study are consistent with *X. cheopis’* seasonal distribution patterns being determined by pre-imaginal development times [30].

Our results point towards the possibility that the endemic *S. fonquerniei* is better adapted to colder conditions found outdoors in the plague-endemic highlands. Our results suggest that a study with a wider range of temperatures and humidities and constant as well as fluctuating climate conditions would add valuable data and improve our understanding of the factors that determine the distribution of plague in Madagascar. In order to understand the vector-pathogen-host dynamics of plague an additional study evaluating the effects of different temperatures and RHs on plague transmission to mammal hosts by these flea species would be extremely valuable.

## Abbreviations

AIC: Akaike Information Criterion
DD: Degree-Days
IPM: Institut Pasteur de Madagascar
LDT: Larval Development Time
PDT: Pupal Development Time
RH: Relative Humidity
*S. fonquerniei*: *Synopsyllus fonquerniei*
*X. cheopis*: *Xenopsylla cheopis*
